# Association of Emergency Department Length of Stay and Crowding for Patients with ST-Elevation Myocardial Infarction

**DOI:** 10.5811/westjem.2015.8.27908

**Published:** 2015-12-16

**Authors:** Michael J. Ward, Olesya Baker, Jeremiah D. Schuur

**Affiliations:** * Vanderbilt University, Department of Emergency Medicine, Nashville, Tennessee; † Brigham and Women’s Hospital, Department of Emergency Medicine, Boston, Massachusetts

## Abstract

**Introduction:**

With the majority of U.S. hospitals not having primary percutaneous coronary intervention (pPCI) capabilities, the time spent at transferring emergency departments (EDs) is predictive of clinical outcomes for patients with ST-elevation myocardial infarction (STEMI). Compounding the challenges of delivering timely emergency care are the known delays caused by ED crowding. However, the association of ED crowding with timeliness for patients with STEMI is unknown. We sought to examine the relationship between ED crowding and time spent at transferring EDs for patients with STEMI.

**Methods:**

We analyzed the Centers for Medicare and Medicaid Services (CMS) quality data. The outcome was time spent at a transferring ED (i.e., door-in-door-out [DIDO]), was CMS measure OP-3b for hospitals with ≥10 acute myocardial infarction (AMI) cases requiring transfer (i.e., STEMI) annually: Time to Transfer an AMI Patient for Acute Coronary Intervention. We used four CMS ED timeliness measures as surrogate measures of ED crowding: admitted length of stay (LOS), discharged LOS, boarding time, and waiting time. We analyzed bivariate associations between DIDO and ED timeliness measures. We used a linear multivariable regression to evaluate the contribution of hospital characteristics (academic, trauma, rural, ED volume) to DIDO.

**Results:**

Data were available for 405 out of 4,129 hospitals for the CMS DIDO measure. These facilities were primarily non-academic (99.0%), non-trauma centers (65.4%), and in urban locations (68.5%). Median DIDO was 54.0 minutes (IQR 42.0,68.0). Increased DIDO time was associated with longer admitted LOS and boarding times. After adjusting for hospital characteristics, a one-minute increase in ED LOS at transferring facilities was associated with DIDO (coefficient, 0.084 [95% CI [0.049,0.119]]; p<0.001). This translates into a five-minute increase in DIDO for every one-hour increase in ED LOS for admitted patients.

**Conclusion:**

Among patients with STEMI presenting to U.S. EDs, we found that ED crowding has a small but operationally insignificant effect on time spent at the transferring ED.

## INTRODUCTION

Timeliness of myocardial perfusion is an important predictor of long-term outcomes for patients with ST-elevation myocardial infarction (STEMI). 1 Since the majority of hospitals in the U.S. do not have primary percutaneous coronary intervention (pPCI) capabilities, 2 many STEMI patients require transfer to pPCI-capable facilities to restore myocardial perfusion. More time spent at transferring emergency departments (EDs) has been shown to be associated with increased mortality for patients with STEMI. 3 Further heightening the importance of the role of the ED and its timeliness is that most patients with STEMI initially present to U.S. EDs. 4 Not only are transfers from U.S. EDs increasing in frequency for patients with STEMI, 5 but the time spent at transferring EDs is longer and more variable than either the transportation or pPCI center phases. Considering the role of the emergency care system is to rapidly identify, coordinate and treat time-sensitive emergency conditions like STEMI, the timeliness and performance of the ED for patients with STEMI is central to high-quality care.

To measure the timeliness of transferring EDs, the American Heart Association (AHA) recognizes a quality measure called the “door-in-door-out” time (DIDO) interval. 6 While no specific time benchmark is endorsed, studies recommend that DIDO should be no longer than 30 or 45 minutes before clinical outcomes are diminished. 3, 7 However, only 10% of transferred STEMIs met the more stringent 300-minute benchmark. 3 Potentially influencing the ability of EDs to meet these timeliness goals is ED crowding. Studies of ED crowding and prolonged ED length of stay (LOS) have found associations with lower quality care. 8 However, the influence of ED crowding and timeliness of patient transfer for STEMI is unknown. Considering that unique policies exist, such as prehospital and triage electrocardiograms (EKGs), and guidelines for timeliness, EDs have developed policies to identify STEMIs in the setting of crowding. Therefore, we sought to quantify the association between ED crowding and time spent at transferring EDs for patients with STEMI in U.S. EDs.

## MATERIALS AND METHODS

We analyzed the Centers for Medicare and Medicaid Services (CMS) hospital quality data from 2012. The primary outcome, DIDO, is CMS measure OP-3b, ED Median Time to Transfer a Patient with acute myocardial infarction (AMI) for acute coronary intervention. OP-3b data included hospitals with ≥10 AMI cases annually. While the term AMI includes a broader group of cardiovascular emergencies, OP-3b only measures those with STEMI and new left bundle branch block requiring acute coronary intervention. 9 We selected four surrogate measures of ED crowding for our analyses. Our primary measure of ED crowding was ED-1: median ED LOS for admitted patients. We hypothesized that ED LOS for admitted patients would explain significant variation in DIDO for several reasons. First, boarding of admitted patients in the ED was identified by the 2006 Institute of Medicine report, Hospital-Based Emergency Care, as a key cause of crowding in the ED. 10 Second, the proportion of ED patients admitted and higher inpatient occupancy rates were associated with increased ED LOS. 11 Last, ED admitted LOS may be more reflective of STEMI ED patient care as the severity of illness for STEMI patients is more comparable to patients admitted from the ED rather than those who are discharged.

Beyond ED admitted LOS, we included additional ED timeliness measures in our analyses: 1) discharged LOS (OP-18b: Median ED LOS for Discharged Patients); 2) boarding time (ED-2: Median time from admit decision time to time of departure from ED for ED patients admitted to inpatient status); and 3) waiting time to be seen by a clinical provider (OP-20: Door to Diagnostic Evaluation by a Qualified Medical Professional).

We identified facilities that were eligible to report OP-3b. Of those that reported OP-3b, we examined the distribution of DIDO times by facility types and facility characteristics. Facility types were obtained from the AHA annual survey and included academic, trauma center, rural/urban location (U.S. Department of Agriculture classification) and number of hospital beds (i.e., bed size). Facility characteristics included ED patient volume, ED admitted LOS, ED discharged LOS, boarding time, and waiting time. We used a bivariate linear regression model to determine the relationship between DIDO and ED crowding measures (admitted LOS, discharged LOS, boarding time, and waiting time). We used a multivariable linear model to evaluate the contribution of hospital characteristics to DIDO. The model assumed normally distributed errors, homoscedasticity, and a low degree of multicollinearity. We also selected the longer of the two DIDO benchmarks, 45 minutes, since few EDs met the more stringent 30-minute benchmark and would be unlikely to be implemented in practice. 3 We tested whether ED crowding measures were affected if EDs met this 45-minute benchmark for DIDO using a two-sample t-test with a level of significance of P<0.05. We conducted all analyses using Stata v13.1 (College Station, TX).

## RESULTS

We identified 405 out of 4,129 hospitals eligible for inclusion with >10 cases; 1,406 had between one and 10 cases to report, 923 had no cases, and 1,395 did not have results available for the reporting period. Included facilities were primarily non-academic (99.0%), non-trauma centers (65.4%), in urban locations (68.5%). Overall median DIDO was 54.0 minutes (IQR 42.0, 68.0). We report DIDO performance by facility characteristics and ED crowding measure (Table 1 and 2).

Of the reporting hospitals, 30.1% (119/396) met the 45-minute DIDO benchmark. In bivariate linear regression analyses of the four ED crowding measures and DIDO, median ED admitted LOS (coefficient, 0.044 [95% CI [0.012–0.076]]; p=0.007) and boarding time (coefficient, 0.043 [95% CI [0.001–0.085]]; p=0.047) were significantly associated with DIDO, while waiting time (p=0.6) and ED discharged LOS (p=0.2) were not associated with DIDO. Hospitals with an average DIDO <45 minutes had a significantly lower median ED admitted LOS than hospitals with DIDO ≥45 minutes (259 vs. 283 minutes; p=0.008). Similarly, median boarding times were significantly lower for those with average DIDO <45 versus those ≥45 minutes (90.0 vs. 105 minutes; p=0.027).

After adjusting for hospital characteristics, a one-minute increase in ED LOS at transferring facilities was associated with DIDO (coefficient, 0.084 [95% CI [0.049–0.119]]; p<0.001). This translates into a five-minute increase in DIDO for every one-hour increase in ED LOS for admitted patients. Among hospital characteristics, urban setting (coefficient, −9.52 [95% CI [−15.6 - −3.47]]; p=0.002), the third (coefficient, −13.3 [95% CI [−24.8- −1.73]]; p=0.024) and fourth (coefficient, −13.7 [95% CI [−26.8 - −0.655]]; p=0.040) highest quartiles of ED patient volumes were associated with shorter DIDO times. The scatterplots of each measure versus DIDO can be seen in the Figure.

We tested model assumptions of linearity, normality, homoscedasticity and correlation. To test for linearity, we plotted the standardized residuals against continuous predictor variables. We did not observe a nonlinear pattern, indicating that the linearity assumption was reasonable. The distribution of standardized residuals was slightly skewed due to the presence of outliers. We determined these outliers to be appropriate data points and decided to include them in the model. We tested for homoscedasticity by plotting residuals against fitted values, which produced a random scatter indicating homoscedasticity. Finally, we checked for multicollinearity using variance inflation factor (VIF). All explanatory variables had low VIF values.

## DISCUSSION

We found that longer DIDO time for STEMI patients requiring transfer for acute coronary intervention was associated with a longer ED admitted LOS. However, an approximately one-hour increase in ED admitted LOS was associated with only a five-minute longer DIDO time – unlikely to be clinically significant as this duration represents approximately 4% of the maximum recommended benchmark. Such a small increase in DIDO for a large increase in ED LOS suggests that crowding may have a minimal effect on an ED’s ability to identify and transfer patients requiring acute coronary intervention.

Since the presentation of individual time-sensitive diseases (e.g., STEMI) can occur infrequently depending upon the ED’s patient volume, measures that capture hourly and daily ED performance for the general ED population may represent more effective measures of a system’s readiness to handle time-sensitive emergencies. Our findings suggest that measures dissimilar to the admitted population (e.g., overall ED LOS) may not reliably reflect timeliness for critical conditions such as STEMI. Moreover, performance measures involving admitted patients may be a better indicator of the quality of time-sensitive conditions (e.g., STEMI) compared with other crowding measures. For example, the boarding time measure captures the ED population who are admitted and waiting for a hospital bed. Considering that hospital congestion can limit bed availability in the ED, the degree of boarding affects not only admitted patients, but those who may be discharged home as well. On the other hand, other measures involving discharged patients and waiting time to see a clinician may not reflect the key process step for rapid diagnosis of a STEMI, namely EKG use. For example, in order to comply with the AHA guidelines for a rapid EKG, 6 EDs have developed evidence-based triage protocols to obtain EKGs on patients with symptoms suggestive of STEMI at arrival prior to clinician evaluation. 12 Concerning EKGs and patients with presentations consistent with STEMI can result in the patient bypassing any triage line and trigger early activation of the transfer process. Patients with a suspected STEMI are therefore likely to have a much shorter waiting time to be seen by an ED clinician as a direct result of these policies. However, patients with suspected STEMI represent a minority of the ED population, and therefore, true waiting times are likely to be much longer.

While we found a minimal influence of ED crowding on transfer timeliness, one finding of our study is that nearly 70% of included EDs did not meet the recommended 45-minute DIDO threshold. This finding is consistent with other studies examining the 45-minute benchmark. 7 This suggests that there is a substantial opportunity to improve the timeliness of transfers for patients with STEMI from U.S. EDs. As other studies have also identified a similarly poor national transfer performance, 3, 13 we recommend enhancing the prominence of transfer performance by publicly reporting the proportion of patients meeting the 45-minute benchmark. Doing so would provide more meaningful data to consumers and for quality improvement efforts.

## LIMITATIONS

Our results should be considered in light of several limitations. As this is an administrative dataset, we did not know the pPCI capabilities of facilities, which may affect the timeliness and decision to transfer. We also did not know the proximity of each facility to pPCI centers; however, rural/urban status is a proxy measure for this facility characteristic. Since these facilities had ≥10 transfers annually, our results are not generalizable to facilities with lower patient volume (i.e., less than 10 transfers). While there were 1,395 facilities that had results unavailable for the reporting period, this likely had minimal effect on our results as eligible facilities face large financial penalties for not reporting measure OP-3b to CMS. Further, this group also includes critical access hospitals that likely care for few patients with STEMI.

## CONCLUSION

Among STEMI patients presenting to U.S. EDs, we found that ED crowding has a small but operationally insignificant effect on STEMI DIDO times. These results suggest that ED performance during the transfer of STEMI patients is minimally affected by ED crowding. As few EDs meet a recommended transfer time of 45 minutes, we propose that CMS report the proportion of STEMI patients who meet recommended DIDO times.

## Figures and Tables

**Figure:**
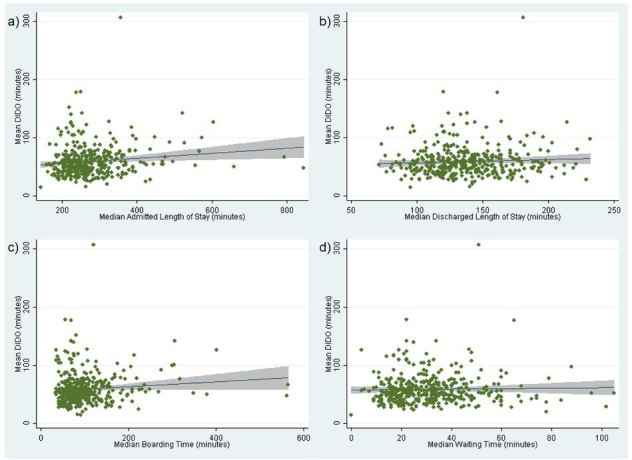
Scatterplots of Door-In-Door-Out (DIDO) times versus emergency department (ED) crowding measures: a) ED admitted length of stay (LOS); b) ED discharged LOS; c) Boarding time; and d) Waiting time. The lines represent the fitted values from the bivariate model and the shaded gray area represents the 95% confidence interval.

**Table 1:** Door-in-door-out (DIDO) performance by hospital characteristics. All times are in minutes.

	N	%	Mean DIDO	Median DIDO	Interquartile range
All hospitals	405	100	59.5	54.0	42.0, 68.0
Academic status					
Academic	4	1.00	71.0	76.0	58.5, 83.5
Non-academic	399	99.0	59.4	54.0	42.0, 67.0
Trauma status					
Trauma	140	34.6	62.2	54.0	43.0, 68.0
Non-trauma	265	65.4	58.1	53.0	42.0, 67.0
Rural/urban status					
Rural	127	31.5	65.0	59.0	45.0, 75.0
Urban	276	68.5	56.9	52.0	42.0, 66.0
Hospital bed size* (by groups)					
1–3	149	37.0	63.2	53.0	43.0, 70.0
4	161	40.0	57.8	55.0	42.0, 66.0
5–8	93	23.1	56.6	52.0	42.0, 68.0
Emergency department yearly volume (quartile)					
Q1: 16–12,772	26	6.72	66.0	54.5	45.0, 84.0
Q2: 12,954–27,420	130	33.6	63.3	55.0	45.0, 68.0
Q3: 27,720–48,812	170	43.9	54.3	52.0	40.0, 63.0
Q4: 49,264–337,128	71	18.1	57.5	55.0	43.0, 70.0

Hospital bed size ranges from the American Hospital Association website: 1) 6–24, 2) 25–49, 3) 50–99, 4) 100–199, 5) 200–299, 6) 300–399, 7) 400–499, 8) 500+.

**Table 2:** Door-in-door-out performance by operational characteristics of emergency department (ED) crowding measures by quartile.*

	N	%	Mean DIDO	Median	Interquartile range
ED admitted length of stay (LOS) (ED-1: median ED LOS for admitted patients)					
Q1: 84–215 minutes	68	17.1	54.0	51.5	38.5, 63.0
Q2: 216–259	139	35.0	60.1	51.0	44.0, 64.0
Q3: 260–314	105	26.4	54.2	54.0	41.0, 64.0
Q4: 316–1,031	85	21.4	66.9	60.0	48.0, 76.0
ED discharged LOS (OP-18b: median ED LOS for discharged patients)					
Q1: 60–112 minutes	82	21.0	56.0	53.0	42.0, 66.0
Q2: 113–135	127	32.5	58.7	53.0	41.0, 65.0
Q3: 136–162	105	26.9	58.6	55.0	43.0, 68.0
Q4: 163–860	77	19.7	62.0	53.0	43.0, 70.0
Boarding time (ED-2: median time from admit decision time to time of departure from ED for ED patients admitted to inpatient status)					
Q1: 0–61 minutes	86	21.7	58.4	51.5	41.0, 67.0
Q2: 62–88	122	30.8	58.2	52.0	43.0, 64.0
Q3: 89–126	110	27.8	58.8	55.5	46.0, 66.0
Q4: 127–584	78	19.7	61.4	59.0	42.0, 73.0
Door-to-diagnostic evaluation (OP-20: door to diagnostic evaluation by a qualified medical professional )					
Q1: 0–19 minutes	90	23.1	55.2	50.0	40.0, 62.0
Q2: 20–28	98	25.2	60.2	56.0	46.0, 66.0
Q3: 29–40	114	29.3	59.2	55.5	45.0, 70.0
Q4: 41–749	87	22.4	60.8	52.0	42.0, 70.0

* Door-in-door-out by ED crowding measures by quartile.
